# Chitosan-GPTMS-Silica Hybrid Mesoporous Aerogels for Bone Tissue Engineering

**DOI:** 10.3390/polym12112723

**Published:** 2020-11-17

**Authors:** María V. Reyes-Peces, A. Pérez-Moreno, Deseada María de-los-Santos, María del Mar Mesa-Díaz, Gonzalo Pinaglia-Tobaruela, Jose Ignacio Vilches-Pérez, Rafael Fernández-Montesinos, Mercedes Salido, Nicolás de la Rosa-Fox, Manuel Piñero

**Affiliations:** 1 Department of Condensed Matter Physics 1, Faculty of Science, University of Cadiz, 11510 Cádiz, Spain; Maria.reyes@uca.es (M.V.R.-P.); antoniopmoreno@gmail.com (A.P.-M.); nicolas.rosafox@uca.es (N.d.l.R.-F.); 2 Instituto de Investigación e Innovación Biomédica de Cádiz (INIBICA), 11009 Cádiz, Spain; gonzalo.pinaglia@uca.es (G.P.-T.); nachovilper@gmail.com (J.I.V.-P.); rafael.montesinos@uca.es (R.F.-M.); mercedes.salido@uca.es (M.S.); 3 Department of Physical Chemistry, Faculty of Science University of Cadiz, 11510 Cádiz, Spain; desire.delossantos@uca.es; 4 Department of Chemical Engineering, Faculty of Science University of Cadiz, 11510 Cádiz, Spain; mariadelmar.mesa@uca.es; 5 Instituto de Microscopía Electrónica y Materiales (IMEYMAT), University of Cadiz, 11510 Cádiz, Spain; 6 Department of Histology, SCIBM, Faculty of Medicine University of Cadiz, 11004 Cádiz, Spain

**Keywords:** hybrid silica aerogels, chitosan, GPTMS, textural properties, mechanical properties, swelling properties, bioactivity, bone tissue engineering, osteoblasts, focal adhesions

## Abstract

This study introduces a new synthesis route for obtaining homogeneous chitosan (CS)-silica hybrid aerogels with CS contents up to 10 wt%, using 3-glycidoxypropyl trimethoxysilane (GPTMS) as coupling agent, for tissue engineering applications. Aerogels were obtained using the sol-gel process followed by CO_2_ supercritical drying, resulting in samples with bulk densities ranging from 0.17 g/cm^3^ to 0.38 g/cm^3^. The textural analysis by N_2_-physisorption revealed an interconnected mesopore network with decreasing specific surface areas (1230–700 m^2^/g) and pore sizes (11.1–8.7 nm) by increasing GPTMS content (2–4 molar ratio GPTMS:CS monomer). In addition, samples exhibited extremely fast swelling by spontaneous capillary imbibition in PBS solution, presenting swelling capacities from 1.75 to 3.75. The formation of a covalent crosslinked hybrid structure was suggested by FTIR and confirmed by an increase of four hundred fold or more in the compressive strength up to 96 MPa. Instead, samples synthesized without GPTMS fractured at only 0.10–0.26 MPa, revealing a week structure consisted in interpenetrated polymer networks. The aerogels presented bioactivity in simulated body fluid (SBF), as confirmed by the in vitro formation of hydroxyapatite (HAp) layer with crystal size of approximately 2 µm size in diameter. In vitro studies revealed also non cytotoxic effect on HOB^®^ osteoblasts and also a mechanosensitive response. Additionally, control cells grown on glass developed scarce or no stress fibers, while cells grown on hybrid samples showed a significant (*p* < 0.05) increase in well-developed stress fibers and mature focal adhesion complexes.

## 1. Introduction

A new generation of bio-based aerogels has been attracted much attention research during the last two decades, particularly in emerging areas associated to environmental and biomedical sciences [1,2,3,4,5]. Thanks to their unique and tuneable properties, as well as ease of functionalization, a widespread range of applications has been proposed and developed for silica-based biopolymer aerogels [6]. Moreover, their characteristic 3D open network structure facilitates the access for external fluids, which allows a controlled interaction with internal active surfaces and/or encapsulated secondary phases. Due to its versatility, these type of materials have been proposed for very different applications, such as catalysis [4], CO_2_ capture [7], oil–water separation [8], drug delivery and medicine [9]. In this context, a well-diversified collection of biopolymers has been considered for the preparation of bio-aerogels, including collagen [10], gelatin [11], whey proteins [12], etc. Also polysaccharides such as alginate [13], cellulose [14], chitosan (CS) [15] and many others have been successfully used to produce bio-based aerogels [16,17]. Among the natural compounds, CS (a biopolymer extracted from chitin natural source) has attracted great interest due to their remarkable properties for industrial technology and biomedical fields [18,19,20].

Besides, extensive research has been conducted on the chemical modifications of chitosan, as summarized in numerous and excellent publications focused on this topic [21,22,23,24] Hence, the hybridization of CS with an inorganic silica network by using sol-gel method has been investigated so far, as a strategy to achieve improved properties from a combined synergistic effect of the properties of the individual organic and inorganic component [15,25]. Moreover, silica is considered to be a decisive part of the mechanism of biomineralization of biomimetic apatite on bioactive surfaces [26], so it may help to stimulate adequate biological response in biomaterials. Regarding this issue, silanol groups in the surface acts a nucleation sites together with factors related with the textural characteristics of the materials, thus controlling the mechanism for nucleation and growth of biomimetic hydroxyapatite layer [27]. Therefore, silica-based hybrid aerogel structures, presenting tunable mechanical and chemical properties, may also improve the primary characteristics of biomaterials: non-cytoxicity, biocompatibility, bioactivity, biodegradability, etc. [28,29]. Consequently, hybrid aerogels in the CS-silica system are expected to improve mechanical strength as well as biological properties of the resulting biomaterials in the field of biomedical engineering.

To date, various CS-silica aerogel synthesis processes have been reported and, in general, all of them involved a mixture of sol-gel precursors followed by the formation of the hybrid gel [25,30,31,32] The subsequent extraction of the pore filling liquids from the wet gels is the most important step for obtaining the hybrid aerogels, which has been usually accomplished by supercritical drying [31,33] However, also freezing drying [34] or ambient pressure drying [25] methods have been successfully employed. The first reported fabrication of CS-silica hybrid monolithic aerogels using CO_2_ supercritical drying was performed by Ayers and Hunt [15], who described the influence of CS on the physical properties and the biocompatibility of the resulting aerogels. After a thorough investigation of the topic, CS-silica aerogels were proposed for different applications and here we will take special consideration of biomaterials for biomedical purposes [1,2,20,26,35,36]

**Figure 1:**
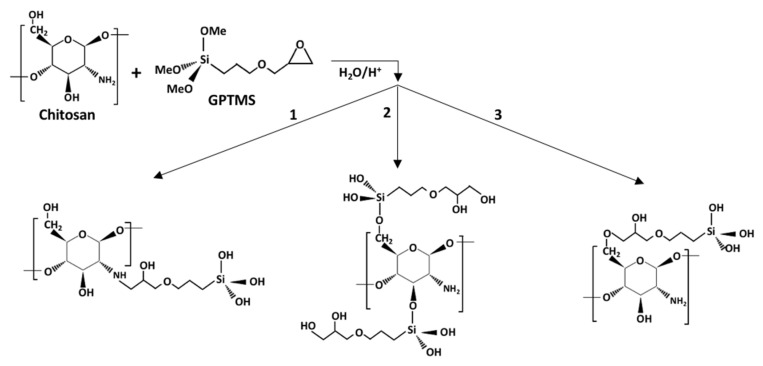
Alternatives routes of functionalization of deacetylated chitosan by GPTMS in acidic medium. (**1**) Trough nucleophilic attack of epoxide ring by amine group of chitosan; (**2**) by condensation of silanol and OH groups of chitosan; (**3**) by nucleophilic attack of epoxide by OH group of chitosan [40].

To this end, many research studies presented in the literature about the synthesis of chitosan-silica hybrids proposed the use of crosslinking agents, to create strong interactions at nanoscale with creation of covalent bonds between chitosan and silica network [37]. This strategy is favored by the presence of both hydroxyl and primary amine groups in chitosan molecule (a heteropolymer made of glucosamine and N-acetyl glucosamine), that facilitates the introduction of coupling agents. Best reported results were observed using 3-glycidoxypropyl trimethoxysilane (GPTMS), one of the most frequently used alkoxysilanes to synthesize class II hybrid materials [37,38] according to the hybrid classification of Gomez-Romero and Sanchez [39] In acidic aqueous solution, GPTMS may proceeds to functionalize chitosan creating covalently bonded compounds, according to different reaction mechanisms (see Figure 1) [40,41]. So, for example, through the nucleophilic attack by the primary amine group of chitosan to open the epoxide ring, (route 1); by condensation of silane groups of GPTMS with hydroxyl groups of chitosan (route 2); through nucleophilic attack by the hydroxyl groups of the chitosan to open the epoxide ring (route 3). In addition, some ionic chemical species can be formed too between positively charged amine groups and negatively charged silanes, or negatively charged oxygen from the epoxide group. In all cases, the methoxysilane groups hydrolyzed to form silanol groups simultaneously, as shown in Figure 1, and may eventually condense with the silica network developed from the corresponding inorganic component of the hybrid, to form the Si–O–Si covalent bonds and thus creating a crosslinked mechanically reinforced structure.

Additionally, several studies have focused on the preparation of CS-silica hybrid materials using tetraethoxysilane (TEOS) as silica source and GPTMS crosslinker, reporting the formation of covalent bonding between silica and CS networks [40,41]. The resulting materials were intended for drug delivery technology [42,43,44] but also as separation membranes in biomaterials [45]. Other authors obtained interpenetrating network hybrid membranes made of silica and CS instead, without using crosslinking agents [46]. Nevertheless, despite significant achievements in the preparation and characterization of CS-silica hybrids for biomedical purposes, and the hundreds of articles related to this topic, more efforts are needed to evaluate the potential applications of their corresponding mesoporous aerogels as biomaterials for tissue engineering.

The current study suggests that chitosan-silica mesoporous aerogels has osteoregenerative properties. To this end, we synthesized monolithic hybrid aerogels by sol-gel followed by supercritical CO_2_ drying, based on chitosan (CS) and two types of alkoxysilanes: TEOS, as silica inorganic precursor, and GPTMS, as crosslinker agent, used in different molar ratios. We prepared several formulations of hybrid aerogels incorporating different amounts of chitosan and GPTMS and examined the effect of their compositions on the structure, mechanical and swelling properties. In addition this work investigates the in vitro bioactivity of the hybrid materials in simulated body fluid (SBF), as well as the osteoblast cell response of the hybrids by in vitro culture methods.

## 2. Materials and Methods

### 2.1. Materials

Tetraethylortosilicate (TEOS, 99%) and Chloride acid (HCl) (37%) were obtained from Alfa Aesar (Haverhill, MA, USA). Chitosan (CS; 50,000–190,000 Da; 75%–85% deacetylation degree) and 3-glycidoxypropyltrimethoxysilane (GPTMS, >98%) were purchased for Sigma Aldrich (St. Louis, MO, USA). Absolute ethanol (99.5%) was purchased from Panreac (Barcelona, Spain). Acid acetic (Reagent Grade) was purchased for Scharlau (Barcelona, Spain). HOB^®^ human osteoblasts, foetal calf serum and Osteoblast Growing Medium (Promocell, Heidelberg, Germany). Paraformaldehyde, PBS, Triton x-100, bovine serum albumin, Metanol, rhodamine phalloidin and monoclonal anti-vinculin FITC conjugate were all purchased from Sigma, (St. Louis, MI, USA), Vectashield^®^ (Vector, Burlingame, CA, USA).

### 2.2. Synthesis of TEOS and CS-GPTMS Precursor Sols

**Figure 2:**
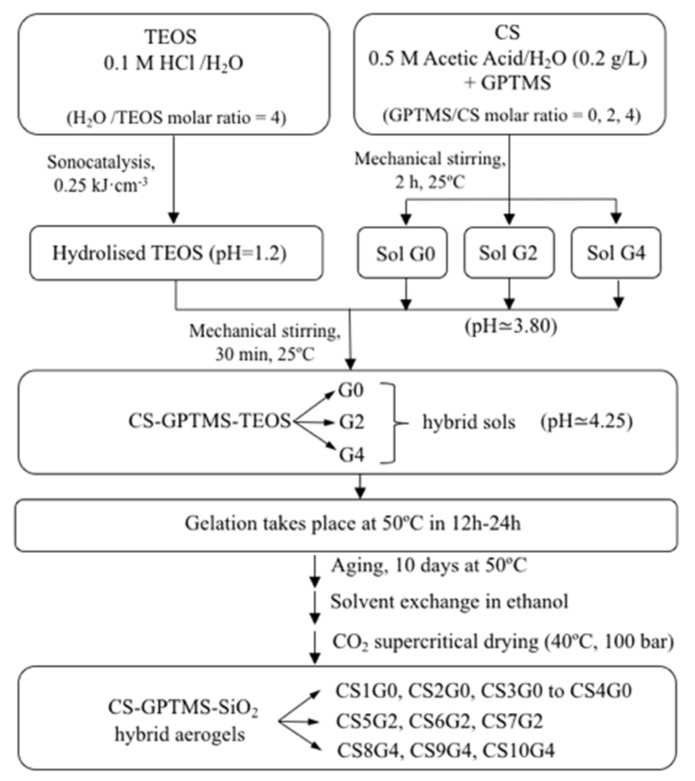
Scheme of the synthesis procedure. In total, ten hybrid aerogels were prepared, denoted as CSnGx (n is a code number that goes from 1 to 10 and x = 0, 2, 4, indicates the GPTMS/CS monomer molar ratio).

CS-silica and CS-GPTMS-silica hybrid aerogels were fabricated with different compositions using the sol-gel method by mixing two precursor sols. The first one made of hydrolysed TEOS with the stoichiometry acid water and the second one was prepared by mixing low molecular weight chitosan and GPTMS (where necessary) in aqueous acetic acid solution. The diagram in Figure 2 summarizes the key steps of the complete synthesis process.

First, TEOS was totally hydrolysed with stoichiometric quantity of 0.1 N HCl under the catalyst effects of ultrasound, by supplying 0.25 kJ/cm^3^ of sonic power using a Vibracell 500 Watt ultrasonic processor from Sonics & Materials (Newtown, CT, USA). A second sol was obtained by dissolving CS powder in 0.5 M aqueous acetic acid solution under vigorous stirring for 2 h at 25 °C to produce 2% *w*/*v* CS solution. Then, different amounts of GPTMS were added drop-wise to this CS solution with 30 min additional mechanical stirring at 25 °C, in order to functionalize the CS biopolymer. In contrast, the preparation of a CS solution without the addition of GPTMS was contemplated. In summary, four sols were obtained: an aqueous silica sol (pH = 1.2) from TEOS, and three additional GPTMS-CS sols with different molar ratios GPTMS:CS monomer: 0 (no GPTMS), 2 and 4 (pH ~ 3.80).

### 2.3. Synthesis of CS-GPTMS-Silica Hybrid Aerogels Monolith

The preparation of hybrid aerogels continued by mixing separately the three CS-GPTMS sols with the hydrolyzed TEOS until homogenization, under mechanical stirring at 25 °C for 30 min. Several inorganic/organic (chitosan) weight ratios and molar ratios of TEOS, CS and GPTMS were examined, as shown in Table 1, where the detailed synthetic conditions used in this work are presented.

The samples with x = 0 were obtained without coupling agent GPTMS, as a reference, being named CSnG0. In total, ten aerogels with several TEOS/CS and GPTMS/CS molar ratios were prepared and characterized. Subsequently, the freshly prepared CS-GPTMS-TEOS sols (pH ~ 4.25) were transferred to 5 mL cylinder-shaped plastic vials and left hermetically closed at 50 °C in an oven, until the gels were set. Following, the resulting alcogels were soaked in ethanol excess at 50 °C for 10 days for aging and removing the residual water from the pore. Finally, wet gels were dried in supercritical CO_2_ at 40 °C and 10 MPa, to obtain the monolithic hybrid aerogels.

### 2.4. Physical and Textural Characterisation

The density of the samples was obtained by measuring the mass and the size of the monolithic cylindrical samples with a sliding calliper and a microbalance (precision ± 0.1 mg). The textural properties of the hybrid aerogels were investigated by means of nitrogen physisorption experiments (Micromeritics ASAP2010, Norcross, GA, USA), working at 77 K and equipped with pressure transducer resolution of 10^−4^ mm Hg). Specific surface area, pore volume and pore size distribution were determined, considering BET and BJH standard models for the analysis. Prior to these experiments, samples were degasified at 120 °C for 6 h.

### 2.5. Thermal Characterization

Thermogravimetric analysis (TGA) was performed with a TGA Q50 from TA Instruments (New Castle, DE, USA) in order to determine the stability of the sample as a function of temperature. The sample heating rate was 10 °C min^−1^ and a temperature ramp of 50 to 900 °C under air atmosphere was used to completely resolved all the weight loss events.

### 2.6. FT-IR Spectroscopy

FTIR was performed on a Bruker Tensor 37 spectrophotometer (Billerica, MA, USA). The spectra were recorded at room temperature with a resolution of 4 cm^−1^ and 100 scans in the region from 500 to 4000 cm^−1^ The samples were stored 24 h at 60 °C, then ground into fine powder, mixed with KBr and pressed into a self-supporting wafer which was put on a sample holder for spectrum measurement.

### 2.7. Swelling Behavior-PBS Swelling Capacity

The swelling ratio of hybrid aerogels was studied to evaluate their swelling behavior and mechanical stability under aqueous medium. To investigate the hybrid network ability to absorb, dry sample monoliths (5 mm high and 8–10 mm in diameter) were immersed into 30 mL phosphate buffer solution (PBS; pH = 7.4) at room temperature. The swelling ratio *SR* of the samples was defined as the weight increase, per unit weight of the original dry hybrid aerogel, due to PBS aqueous solvent absorption. It was calculated in grams of the liquid absorbed PBS per grams of dry sample using Equation (1)

(1) $$SR=\;\frac{W_{t}-W_{d}}{W_{d}}$$

where *W_d_* and *W_t_* correspond to the weights of the sample in dry and in swollen states at time *t*, respectively. Experiments were performed in triplicate using a 30 mm inner diameter basket made of 2 mm aperture stainless steel wire mesh, which was carefully dried for each measurement. Weight measuring was carried out by first blotting both the aerogel surface and the wire basket with filter paper, to remove excess surface PBS, and then weighed immediately. The process was repeated several times at different time intervals, until the sample saturation point *W_∞_* was reached and did not indicate any weight change.

### 2.8. Swelling Kinetics

Besides quantifying the swelling capacity effects for the hybrid aerogels, the knowledge of the swelling kinetics can also be important to evaluate their applicability as porous materials for tissue repair, as it gives information on the rapidity for filling and repairing some bone defects for certain applications [43]. Hence the investigation of the swelling rate for hybrid aerogels should be accomplished. To this end, the absorption behavior was described by the normalized absorption ratio M*(*t*) and was plotted versus √*t.* This square-root-of-time dependency suggested denotes a Lucas-Washburn model for absorption kinetics [47], typical of fast imbibition of liquids by capillary rise, considering a rigid porous structure. The Equation (2) was used:

(2) $$M^{*}\left(t\right)=\frac{W_{t}-W_{d}}{W_{\infty }}$$

where *W_∞_* is the weight of saturated sample.

### 2.9. Mechanical Properties

Mechanical properties of samples were characterized by uniaxial compression (Shimadzu AG-I Autograph, Kyoto, Japan) with a load cell of 5 kN for dry samples and of 50 N for samples immersed in PBS. Cylindrical samples 16–20 mm high and 8–10 mm in diameter were used, fulfilling with ASTM D7012 (h = 2D). The compressive strength and maximum strain were obtained from the maximum deformation before the fracture of the sample, and the elastic modulus from the initial tangent of the stress-strain curve.

### 2.10. In Vitro Bioactivity

Biomineralization was studied by submerging 5 mm length × 8 mm diameter aerogel pellets in 30 mL simulated body fluid SBF in polyethylene containers, through the analysis of hydroxyapatite (HAp) formation at the surface. SBF was prepared according to Kokubo’s method [48] and soaking was maintained for 21 days at 37 °C. The test was performed with fluid weekly exchange and samples were taken out from the buffer solution after every 7 days, carefully washed with Milli-Q (MilliporeSigma, Burlington, MA, USA) for removing surface minerals and later again dried at 50 °C and ambient pressure. Surface morphologies of SBF treated samples after different soaking periods were investigated with SEM/FEI Nova NanoSEM 450 (FEI, Morristown, NJ, USA); (resolution 1.4 nm) equipped with a Bruker SDD-EDS detector, used for determining Ca/P compositional differences across the specimen surface. High resolution imaging HRTEM was performed with TEM TALOS FX200 (Thermo Scientific, Waltham, MA, USA) and selected-area electron diffraction (SAED) patterns were obtained in microprobe mode, in order to identify the crystalline nature of possible nanocrystalline phases.

### 2.11. In Vitro Biocompatibility

HOB^®^ human osteoblasts (Promocell, Heidelberg, Germany) were seeded at a density of 15,000 cells/cm^2^ and incubated in Osteoblast Growing Medium supplemented to a final concentration of 0.1 mL/mL of fetal calf serum (Promocell) at 37 °C and 5% CO_2_ on test surfaces and immunolabelled after 48 h, 72 h and 1 week. Growth medium was changed every two days. HOB cells did not exceed ten population doublings. Aerogels were sterilized in autoclave in order to achieve optimal sterilization, prior to cell seeding. At least five samples of each type were seeded and analyzed per experiment. The test groups for selected samples with higher CS content were as follows: CS4G0, CS7G2 and CS10G4. HOB^®^ cells grown on glass were used as control.

#### 2.11.1. Cell Morphology and Spreading

Cells were daily examined with the phase contrast microscope in order to evaluate cell morphology, alignment and initial adhesion phase to surfaces. Morphological changes, cell distribution and spreading were assessed prior to immunolabelling for fluorescence and CLSM examination of the CS4G0, CS7G2 and CS10G4 samples.

#### 2.11.2. Actin Cytoskeletal Organization and Vinculin Expression

At the end of each experiment, cells were washed with prewarmed phosphate buffered saline (PBS), pH 7.4, and fixed with 3.7% paraformaldehyde at room temperature, washed, and then permeabilized with 0.1% Triton x-100). After washing, cells were preincubated with 1% bovine serum albumin (Sigma) in PBS for 20 min prior to cell immunolabelling for actin cytoskeleton with rhodamine phalloidin (Sigma) and monoclonal anti-vinculin FITC conjugate (Sigma). After 20 min. TiO_2_/PLGA-10, TiO_2_/PLGA-3, TiO_2_/PLGA-100 samples were rinsed with prewarmed PBS prior to mounting with Vectashield ^®^ (Vector, Burlingame, CA, USA).

#### 2.11.3. Confocal Examination

Samples were visualized using an Olympus confocal microscope (Tokyo, Japan). At least five samples were analyzed for each group to assess surface influence on cytoskeletal organization, focal adhesion number, and development and cell morphology. Images were collected and processed using imaging software. At least 50 cells per sample were analyzed. Samples were exposed to the lowest laser power that was able to produce a fluorescent signal for a time interval not higher than 5 min to avoid photobleaching. A pinhole of 1 Airy unit was used. Images were acquired at a resolution of 1024 × 1024, mean voxel size of 209.20 nm.

#### 2.11.4. Image Analysis

To analyze the differences in focal adhesion number between different sample groups, images were collected as frames obtained at 40× magnification and processed using Image J software. All experiments were repeated in triplicate, unless otherwise stated. All data were SPSS analyzed and expressed as mean ± standard deviation. For the variable number of contacts, a descriptive analysis was used to summarize the number of contacts in each experimental group, and a two-way ANOVA and Tukeys comparison of means were employed. Statistical significance was defined as *p* < 0.05.

## 3. Results and Discussion

### 3.1. Synthesis of CS-GPTMS-Silica Hybrid Aerogels

CS-SiO_2_ and CS-GPTMS-SiO_2_ monolithic hybrid aerogels were here synthesized by sol-gel techniques. The specimens were elastic and mechanically resistant upon handling and homogeneous throughout the bulk, suggesting a well distribution and incorporation of the chitosan and silica components, as shown in Figure S1 of Supplementary materials. The influence of both CS and GPTMS on the structure, mechanical and biological activity of the hybrids was studied.

The suggested reaction mechanisms between CS and GPTMS were previously described in Figure 1 and as result of the crosslinking reaction for G2 and G4 aerogel series, a class II hybrid copolymer network was produced [40]. In its place, hybridization without the silane-coupling agent (G0 aerogel series) could lead to the formation of interpenetrating network hybrid aerogels, featuring class I hybrid structure [39], with rigid silica 3D mesoporous structure and swellable CS moieties. Under these conditions, CS would even get involved in condensation reaction with silanol groups to create Si-O-C bonds with carbonyl groups of the polysaccharide.

Code samples and theoretical compositions are shown in Table 1. The remaining content for CS at the end of the synthesis process is also listed and was calculated based on nitrogen Elemental Analysis (EA) data, obtained from dry aerogels. Although CS nominal content ranged from 3.7 to about 16.7 wt% loading, the results show that only a fraction remained at the end of the synthesis. As consequence, the final composition of the hybrids was not well controlled by the amount of CS, due to its dissolution and leaching in the acid media. Additionally, the tendency of GPTMS to diol formation in acid aqueous media, resulting in deactivation of its cross-linking capacity, was also responsible for the observed decreasing CS contents respect to their theoretical compositions [41]. Though, it was possible to obtain a series of hybrid aerogels with different CS and GPTMS concentrations, providing us to study the influence of both components on the structural and mechanical properties of the hybrids.

### 3.2. Bulk Density and Textural Properties

**Figure 3:**
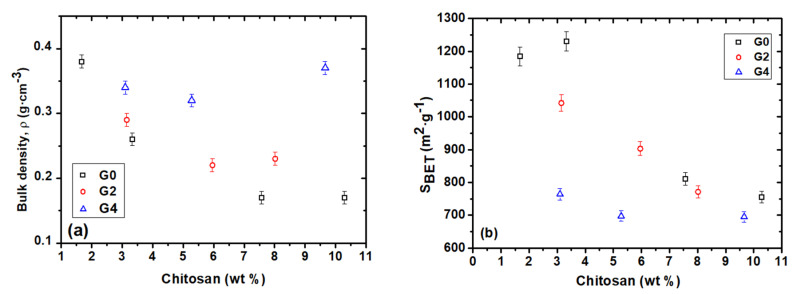
(**a**) Bulk density and (**b**) BET surface area of the CS-GPTMS-silica hybrids as a function of CS content (wt%) obtained from EA.

We estimated the bulk densities of the samples for different CS and GPTMS contents by using geometric measurements. Table 2 and Figure 3a displays the corresponding values for all of the samples studied. A decreasing trend in the density of G0 aerogels series, from the *ρ* = 0.38 g cm^−3^ of CS1G0 sample (CS 1.7 wt%) to the *ρ* = 0.17 g cm^−3^ for both CS3G0 CS (7.6 wt%) and CS4G0 (CS 10.30 wt%) samples, reflects macroscopically the progressive incorporation of the chitosan. For the G2 aerogels series the same decreasing trend in density can be observed. However, the corresponding density values showed a narrower variation range, from *ρ* = 0.29 cm^3^ g^−1^ for CS5G2 (3.1 wt% CS) to *ρ* = 0.22 cm^3^ g^−1^ for CS6G2 (5.9 wt% CS), respectively. These changes reveal the cross-linking action of GPTMS which contributes to the regularization of the hybrid structure. This normalization effect provided by the cross-linker is also observed in the G4 samples series but, in this case, the amount increase of CS determines a slight increase in bulk density, which goes from 0.31 cm^3^ g^−1^ for CS8G4 (3.1 wt% CS) up to 0.37 cm^3^ g^−1^ for CS10G4 (9.7 wt% CS) samples.

Besides, as a consequence of the CS addition and also that of the GPTMS cross-linker, the hybrid aerogels tend to feature lower BET surface area, varying from 1230 m^2^ g^−1^ (CS2G0) to above 700 m^2^ g^−1^ (CS10G4) (Figure 3b). Thus, it was found that aerogels with higher crosslinker content (CS8G4, CS9G4 and CS10G4) presented the lowest surface area values. However, although pore volume and pore size data did not follow an uniform trend, it can be concluded that the gradual inclusion of chitosan inside the porous volume of the silica porous matrix (from 1.7 wt% to above 8–10 wt%), joined to an increase of the crosslink density, produced a reduction of the porous volume from 2.6 cm^3^ g^−1^ (CS1G0) to 1.7 cm^3^ g^−1^ (CS10G4). Meanwhile, pore size experimented a slight increase from 8.1 nm to 11.1 nm for G0 aerogel series, 11.2 nm–14 nm for G2 series and 8.7 nm to 9.8 nm for G4 series. In addition, the porosity was calculated following Equation (3):

(3) $$\%\;porosity=\left(1-\frac{\rho_{bulk}}{\rho_{skeleton}}\right)\times 100$$

where $\rho_{bulk}$ is the bulk density and $\rho_{skeleton}$ is the density of a silica matrix. (2.09 g cm^−3^) [49]. The corresponding porosity values ranged from 81.9% to 91.6% for CS1G0 and CS4G0 samples, respectively, and are presented in Table 2, together with the most relevant values of the structural parameters.

N_2_-physisorption isotherms of CS1G0, CS4G0, CS7G2 and CS10G4 selected samples with 1.7, 10.3, 8.00 and 9.7 wt% CS content, respectively, are shown in Figure S2 (in the Supplementary Materials). We found that this selection of low and high organic and GPTMS content, was representative for describing the structural properties of the obtained aerogels. According to the IUPAC classification all of these samples exhibited type IV isotherms, with a hysteresis loop that determine the presence of interconnected network of mesopores [50], by which multilayer adsorption as it proceeds through capillary condensation process.

A type H1 hysteresis loop, characteristic of materials with uniform pores and narrow pore size distributions (PSD), according to previous structural studies on silica aerogels [51] is observed for the CS1G0 isotherm curve. A change to a type H2 hysteresis loop (CS4G0, CS7G2 and CS10G4 aerogels) was observed by the concurrence of a plateau at high relative pressure (0.9–0.95). This is a sign of the existence of a highly interconnected complex pore network hybrid structure and as a result the pores have a large range of size and shape (Figure 3b). However, as expected, CS1G0 shows a narrow PSD as corresponds to an almost pure silica aerogel (see inset in Figure S2b in the Supplementary Materials).

Also, the well-defined plateau in the CS10G4 isotherm leading to a steep desorption towards low relative pressures is indicative of a desorption process that takes place over only a small pressure range for all pores. However, this plateau is not so well defined for the CS4G0 and CS7G2 isotherms, leading to a further gradual increase of amount adsorbed at high relative pressure (see inset of Figure S2a). This is consistent with the onset of capillary condensation at higher pressures for wider mesopores, as can be seen in Figure S2b (Supplementary Materials) that depicts the PSD obtained by BJH analysis of the desorption branch of the isotherms.

### 3.3. Fourier Transformed Infra-Red (FTIR) Spectral Analysis

**Figure 4:**
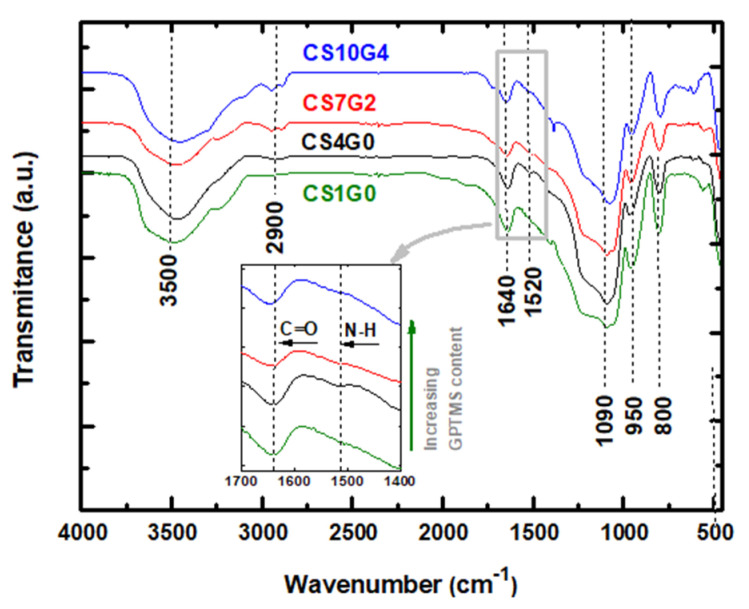
FTIR spectra of CS1G0, CS4G0, CS7G2 and CS10G4 hybrid aerogel samples.

The FTIR spectrum of the CS1G0, CS4G0, CS7G2 and CS10G4 hybrid aerogels showed seven major peaks corresponding to the following wavenumbers 800, 950, 1090, 1520, 1640, 2900 and 3500 cm^−1^ as shown in Figure 4 and their bands’ descriptions will be described below. FTIR spectra showed an intensive broad absorption band located at 1090 cm^−1^ showing intensity increase due to the absorption from the Si–O–C bonds, and a peak at 800 cm^−1^, associated to strong Si-O-Si stretching vibrations. Another band is positioned at 950 cm^−1^ featuring the non-bridging oxygens (Si-OH bonds) in the silica network [40,52,53]. This Si-OH band was most prominent in aerogels without GPTMS, (CS1G0 and CS4G0) associating silanol groups with TEOS precursor rather than with the crosslinker. The absorption peak centered at about 3400 cm^−1^ is assigned to free or adsorbed water and is accompanied by the peak observed at 1640 cm^−1^, a characteristic peak for the Si-OH bond, accounting for the hydrophilic behavior of the silica aerogels [52,53]. Then, its relative intensity decreases as GPTMS content increases, as shown in Figure 4.

Considering the covalent crosslinking between CS and silica network through silane-coupling agent GPTMS the region of interest is found in the range of 1660–1500 cm^−1^ [40,41]. Chitosan powder has two absorption bands in this region: one at 1659 cm^−1^ due to the C=O stretch of the secondary amide in acetylated units of chitosan and a second one small band at 1589 cm^−1^, related with N–H bending of the primary amine in deacetylated units [54,55]. Due to the protonation of chitosan in acidic conditions both of these bands appeared, for the hybrid aerogel samples, slightly shifted from their original position, at 1640 cm^−1^ and 1520 cm^−1^, respectively (41) (see inset Figure 4). Moreover, overlapping of the the C=O amide band at 1640 cm^−1^ with Si-OH bond from silica network, makes it difficult to discern the mechanisms proposed for the formation of covalent links between functionalized chitosan and the silica network. However, the FTIR spectra from the studied samples exhibited a decrease of both amide bands with increasing GPTMS content at 1640 cm^−1^ and 1520 cm^−1^ (inset in Figure 4). Although these FTIR analyses are not conclusive to clearly discern the mechanisms proposed for the coupling between chitosan and GPTMS, these data would suggest the formation of N-C bonding between chitosan and GPTMS. So far, there exists several mechanisms proposed for the coupling between chitosan and GPTMS implying the presence of different types of covalent bonds between the silica matrix and the functionalized chitosan [40].

### 3.4. Thermogravimetric Analysis (TGA)

The thermal stability and degradation behavior of the CS-GPTMS-Silica samples were evaluated by TGA under air atmosphere (see Figure S3; Supplementary Materials). Normally, the hybrids presented enhanced thermal stability for samples with high GPTMS content [25]. The TGA thermograms for the four hybrids CS1G0, CS4G0, CS7G2 and CS10G4 are relatively similar and show three steps of thermal degradation [24,53]. The first step started from above 60 °C to 130 °C. The peak at the DTG at above 90 °C (see inset in Figure S3; Supplementary Materials) showed an initial dehydration weight loss of 7.55%, referring to the physically adsorbed water surface of the polymer. This weight loss decreased with the increasing content for both chitosan and GPTMS. The second weight loss step was found between 130 °C and 220 °C with maximum peak of 195 °C in the DTG curve and corresponding mass losses increased from above 1.2% to 3.5% with increasing the chitosan content in the hybrid samples and it is referred to dihydroxylation of the silanols on the surface, (loss of chemically bound water) and decomposition of chitosan [54]. The third step of weight loss was subdivided in two parts, a first rapid and a second slow weight loss. First rapid weight loss appeared well differentiated between samples with and without GPTMS, and corresponds to the decomposition of the hybrid chains for G2 and G4 hybrids, between 220 and 500 °C, with maximum DTG peak at 260 °C, and of the polysaccharide structure for samples without GPTMS (G0) [24,53,56].

Second slow weight loss occurred between 450 and 800 °C reveals the complete decomposition of the organic components of the hybrids. For the first part, weight losses of 8.8% and 13.5% for CS1G0 and CS4G0 were observed, while 12% and 16% were estimated for CS7G2 and CS10G4, respectively. The second part for slow weight loss was above 4% and 5% for CS7G2 and CS10G2, and around 2% in the case of both CS1G0 and GS4G0 samples, without any range of weight stabilization.

### 3.5. Swelling Behaviour-PBS Absorption

**Figure 5:**
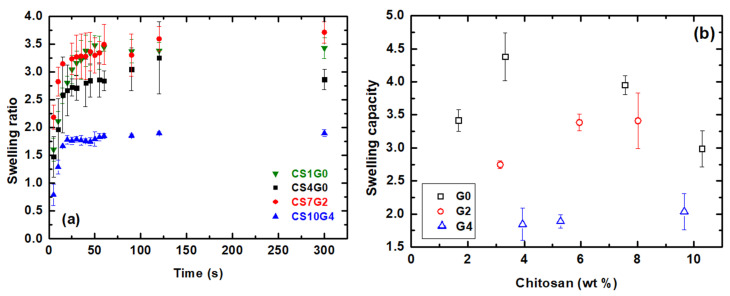
(**a**) Swelling ratio of CS1G0, CS4G0, CS7G2 and GS10G4 aerogels during liquid PBS absorption (**b**) Absorption capacity of CS/GPTMS-SiO2 hybrid aerogels in PBS.

The ability of hybrid samples to absorb phosphate buffered saline (PBS) solution was examined and the swelling curves for selected samples are reported in Figure 5a. All the experiments were done in triplicate to ensure reproducibility. As a general rule, it was observed that increasing the concentration of GPTMS decreases the swelling capacity [57] thus the aerogels presented total absorbed mass ratios ranging between 3.75 and 1.75 for CS7G2 and CS10G4, respectively. It is worth to note that these two samples were mechanically stable in the swollen state, exhibiting characteristic of class II hybrids. Instead, for CS1G0 and CS4G0 (class I hybrids), the swelling process provoked the appearance of multiple cracks at swelling equilibrium although they showed similar swelling capacities of 3.50 and 2.75, separately. These results highlights the importance of the mechanical control during swelling, to develop technical applications based on these samples [32,58]. These features will be studied in more detail in Section 3.7.

Figure 5b represents the swelling capacity versus CS content for different GPTMS/CS molar ratios and confirmed the influence of GPTMS on the swelling capacity, with G4 aerogel series (samples CS8, CS9 and CS10) showing more regular and lowest total absorption values (1.85–2.04). This was explained by the fact that increasing GPTMS made pore size to decreased (see Table 2) and the aerogel became more compact and highly crosslinked, which hinders swelling [32,57]. Summarizing, the affinity of the hybrid chains of the porous aerogels to the aqueous PBS, together with the swelling phenomenon observed in the hybrid structure, would be responsible for these high values of the absorption capacity obtained.

### 3.6. Absorption Kinetics

**Figure 6:**
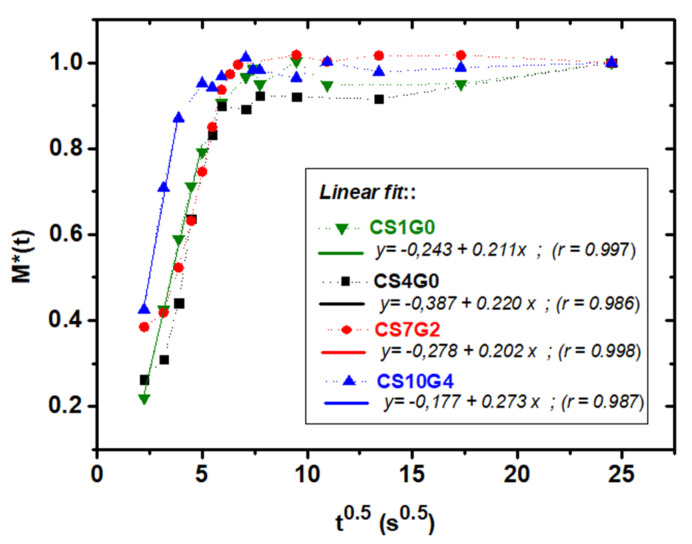
Normalized absorbed mass ratio for CS1G0, CS4G0, CS7G2 and GS10G4 aerogels during liquid PBS absorption versus square-root time. Lines correspond to linear fittings according to standard model of mass increase by imbibition due to capillary rise.

The absorption behavior was described by the normalized absorption ratio M*(*t*) vs. √*t*. It was found that the variation of the sample mass showed a root dependency of t, typical of fast imbibition of liquids by capillary rise, considering a rigid porous structure [51,59]. Also, it was observed that imbibition occurs almost instantaneously and is followed by a final regime toward saturation. The four representative curves for selected samples CS1G0, CS4G0, CS7G2 and CS10G4 are depicted in Figure 6 and all of them followed the √*t* classical-time dependence.

The linear fitting for each curve up to saturation shows well-defined initial linear regimes with similar slopes, revealing similar nanostructures and chemical composition of the porous network, with a slight steeper linear regime for CS10G4, indicating faster absorption, which must be related with its high crosslinking density. Further studies of the kinetic dependence of the absorption with the composition of these hybrids are in progress in order to completely resolve the absorption kinetics of the system.

### 3.7. Mechanical Properties-Uniaxial Compression

The mechanical properties of the hybrids were analyzed on the samples before (dry) and after (wet) the PBS liquid absorption experiments. The uniaxial compression experiments provided an overall vision of the mechanical behavior of the hybrids, and the results obtained are summarized in Table 3, where Young’s modulus, compressive strength and maximum compressive strain are reported. Stress-strain curves from rupture tests under uniaxial compression on selected samples CS1G0, CS4G0, CS7G2 and CS10G4 are plotted for dry (Figure S4a; Supplementary Materials) and wet samples (Figure S4b; Supplementary Materials). First, the stress-strain curves for dry states denoted the existence of two well-differentiated mechanical responses between samples incorporating the GPTMS and samples without the crosslinker.

In general, the increase in CS content is accompanied by a reduction in the elastic modulus and by structural strengthening of the dry aerogels [32]. At the same time, larger deformations were developed before rupture, indicating a brittle-ductile transition through elastomeric behavior, being more evident for higher GPTMS contents. An exception in Young’s modulus progression is CS10G4, where the synergistic effects between the organic and inorganic hybrid components offers the highest compressive strength (96 MPa) and stiffness (50 MPa for Young’s modulus) of all of the hybrid samples. Overall, these results indicate that a large increase in both, the compressive strength (more than 10-fold) and the maximum strain (more than 25-fold), was achieved for CS10G4, respect to previously reported for related samples [41].

More specifically, CS1G0 aerogel behaved as brittle material, showing relatively high elastic modulus (11.2 MPa) and low compressive strength (1.0 MPa) and maximum strain values (12.2%). Increasing CS from 1.7 to 10.3 wt% (CS4G0) allowed to reach up to 50–60% strain (inset Figure S4a; Supplementary Materials). Instead, samples incorporating GPTMS in their chemical structures (G2 and G4 aerogels) showed a rubber-like stress-strain behavior, with high elongation at break and non-linearity as its most obvious feature, typical of synthetic elastomers (CS7G2 and CS10G4 samples).

On the other hand, the uniaxial compression experiments performed under liquid absorption (Figure S4b; Supplementary Materials) led to degraded network structures which, in all cases, behaved as extremely soft materials. Young’s modulus values were in the range (0.16–1.13) MPa and compressive strength between (0.07–0.27) MPa, showing two-three orders of magnitude lower than for dry samples. Nonetheless, hybrids with GPTMS at the rupture strain point in wet state, preserved more or less their original morphology, while samples without crosslinker resulted dispersed in small pieces in the liquid after fracture.

### 3.8. In Vitro Bioactivity Experiments

**Figure 7:**
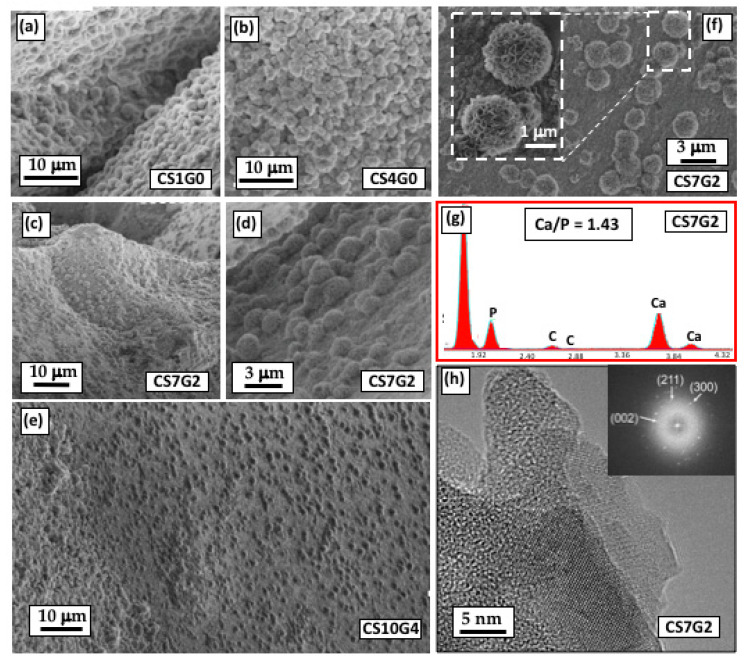
SEM micrographs of the apatite formed on the surfaces of the following hybrid aerogels soaked in SBF for 21 days: (**a**) CS1G0; (**b**) CS4G0; (**c**) CS7G2; (**d**) magnification of precedent micrograph for CS7G2; (**e**) CS10G4; (**f**) CS7G2; (**g**) EDS spectrum from CS7G2 from which Ca/P molar ratio 1.43 was found; (**h**) HRTEM micrograph of CS7G2 and corresponding SAED pattern (inset).

Figure 7 shows SEM micrographs of some hybrids exhibiting bioactivity. All of the samples displayed the precipitation of an apatite-like on the surfaces of the samples about 21 days after immersion in SBF in form of small spherulites. Physiochemical characterization of HAp was performed by using three different techniques: 1. SEM, to observe the characteristic morphology of microcrystals; 2. Elemental EDS analysis, to determine Ca/P compositional variations across the specimen surface; 3. FFT from HRTEM-SAED images, to confirm the crystalline nature of samples.

First, it was observed that spherulites started to precipitate on the aerogels about 7 days after the samples were soaked in SBF, becoming larger and more abundant with increasing soaking time. This mineral phase was formed through heterogeneous nucleation that take place at preferred nucleation sites on the surface of aerogels, forming a layer that recovered it more or less within 21 days soaking, as shown in the micrographs. Thus, Figure 7a,b show surfaces of hybrids CS1G0 and CS4G0 respectively, almost totally covered with HAp spherulites about 2 µm size.

The presence of GPTMS in CS7G2 hybrid provided higher crosslinking density and a more compacted surface. Nonetheless, it also showed a good bioactive response, with HAp recovering all the irregularities of the surface area (Figure 7c), and growing from many surface nucleation sites, as detailed in Figure 7d. Furthermore, CS10G4 aerogel, showing the highest crosslinking density, behaved similarly to the CS7G2, but the surface density of precipitates was the lowest observed in all of the studied samples (Figure 7e), signifying a weak bioactive response in the same soaking time. Otherwise, CS7G2 also showed regions whose surface was not totally recovered by HAp spherulites. (Figure 7f). Higher-magnification micrograph of the HAP crystals formed on CS7G2 soaked in SBF for 21 days showed that the crystal morphology of spherulites consists of a large number of petal-like crystals with the typical cauliflower structure (inset Figure 7f). According to the EDS analysis performed on the CS7G2 sample, the growing mineral phase is calcium-deficient with a Ca/P ratio of 1.43 (Figure 7g). This ratio is expected to increase by simply extending the soaking time to 1 month, desirably reaching values in the range 1.50–1.67 where most biological apatites are usually found.

TEM-EDX and selected area electron diffraction (SAED) pattern confirmed these results. Hence, HRTEM micrograph (Figure 7h) of CS7G2 sample revealed the existence of nanocrystals randomly distributed in the hybrid amorphous matrix, in the size range of about 10–15 nm, probably formed in early stages of the biomineralization of biomimetic apatite. These observations suggest the existence of active surface sites (Si–OH) which promoted nucleation and growth of diverse calcium phosphates, mainly HAp. TEM-EDX microanalysis supported this hypothesis, giving Ca/P ratios between 1.57 and 1.99 from different nanocrystals. Additionally, the small area electron diffraction (SAED) pattern from HRTEM image (inset of Figure 7h) shows bright spots according with the presence of calcium phosphate nanocrystals. In this sense, the d-space for (211), (300) and (002) planes were identified (e.g., d-space for (211) sample was 0.280 nm, in good agreement with standard JCPDS (09-0432) files of HAP). From these observations, it can be concluded that the formed crystals are HAp particles in the precipitation and dissolution process of the apatite layer.

### 3.9. Osteoblast Response In Vitro

#### 3.9.1. Cell Morphology and Spreading

**Figure 8:**
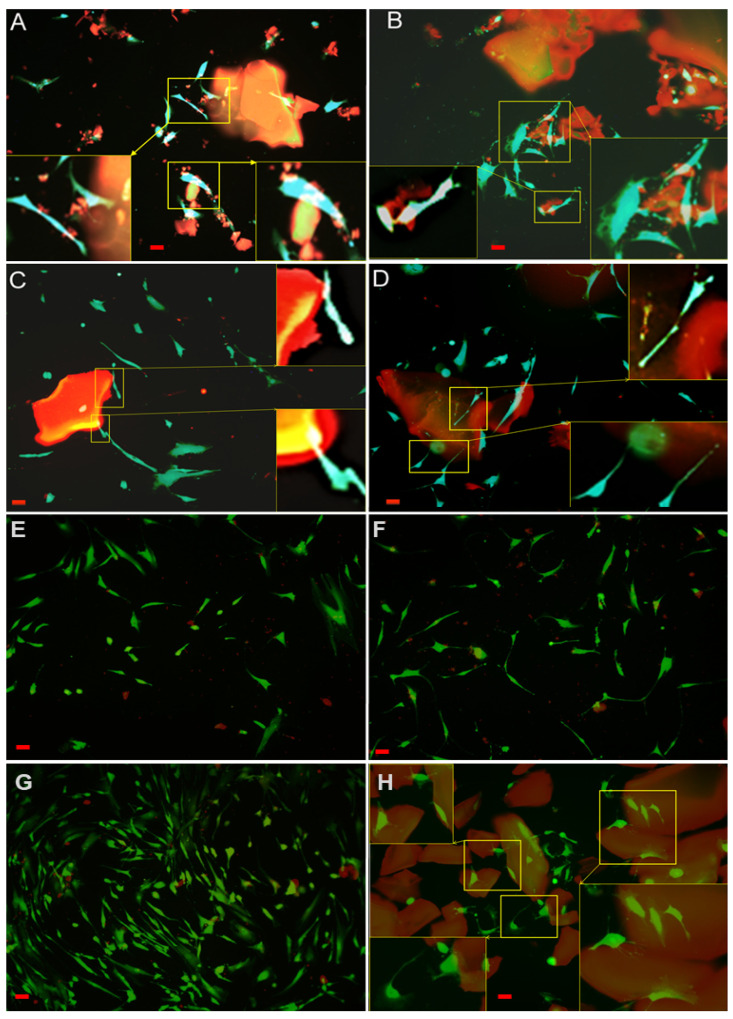
Live/Dead staining of osteoblasts growing on (**A**): CS4G0 after 48 h in culture; (**B**): CS4G0 after 72 h in culture; (**C**): CS7G2 after 48 H in culture; (**D**): CS7G2 after 72 h in culture; (**E**): Control cells grown on glass after 48 h in culture; (**F**): Control cells grown on glass after 72 h in culture. In (**G**): Control cells grown on glass after 1 week in culture; (**H**): osteoblasts grown on CS10G4 after 1 week in culture. Live cells appear green; nuclei of dead cells fluoresce red once examined with fluorescence microscope (10× objective lens). Scale bar represents 50 µm. Squares select and amplify filopodial emissions pointing to material surface.

Attachment, cell growth and phenotypic changes of osteoblasts grown in vitro appeared to be substantially better in cells grown on CS7G2 or CS10G4 samples than in cells grown on the bare substrata, and revealed a successful cell attachment with marked morphological changes, like filopodial and lamellipodial emission, and an improved cell spreading. When experimental groups were compared for the variable number of cells, significant differences were found at initial experimental times (48, 72 h) between CS7G2 and control (*p* = 0.007) and between CS7G2 and CS10G4 (*p* = 1.38 × 10^−5^), and also when cell counts at 48 h and 72 h counts (*p* = 2.57 × 10^−6^) or 48 h and 1 week counts were compared (*p* = 8.97 × 10^−4^). No significant differences in cell number were found after 1 week in culture when control and experimental groups were compared and, instead, significant differences were found in differentiation features related to cell migration and adhesion. Due to the presence of aerogels, osteoblasts developed filopodia and lamellipodia as markers of cell migration that are absent in non-coated groups. Furthermore significantly more efficient focal adhesion sites and stress fibers appeared in the experimental groups. Live/dead staining revealed that the majority of cells were in a viable state (green) at all time points, with only a few dead cells (red) as shown in Figure 8.

**Figure 9:**
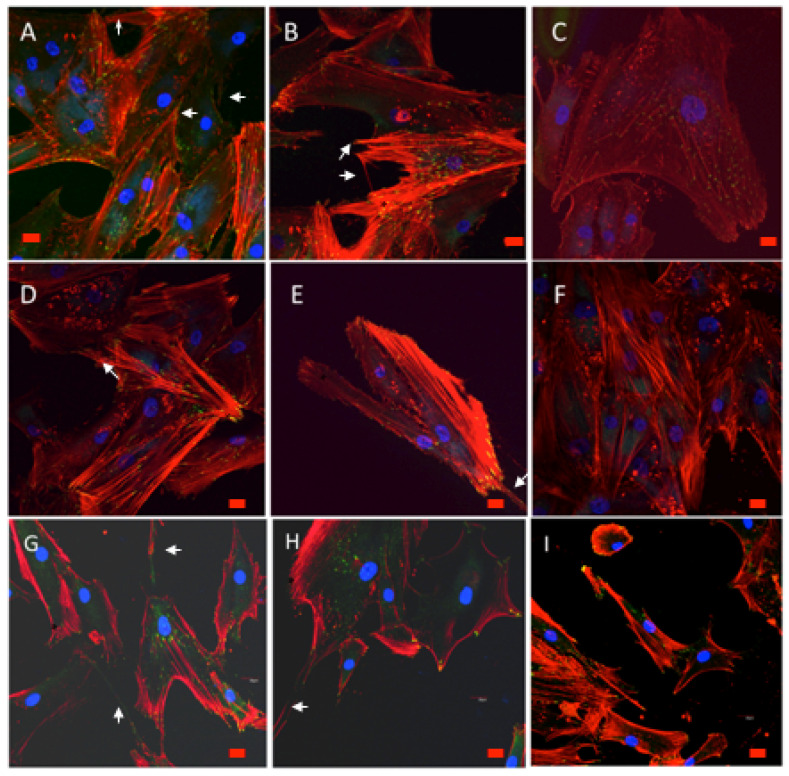
HOB^®^ osteoblasts grown on CS7G2 sample after 48 h (**A**,**B**), 72 h (**D**,**E**) and 1 week (**G**,**H**) in culture, and examined with confocal microscope (40x objective lens). Osteoblasts grown on glass were used as reference control, shown in (**C**), for 48 h in culture, (**F**) after 72 h in culture and (**I**) after 1 week. In red, rhodamine–phalloidin immunolabelled actin cytoskeletal fibers, showing polarization to material and actin cytoskeletal arrangement into stress fibers. Focal adhesions (yellow) were immunolabelled with antivinculin antibody. Nuclei (blue) were DAPI labelled. Arrow marks filopodial emissions, star marks lamellipodia. Scale bar represents 20 μm.

**Figure 10:**
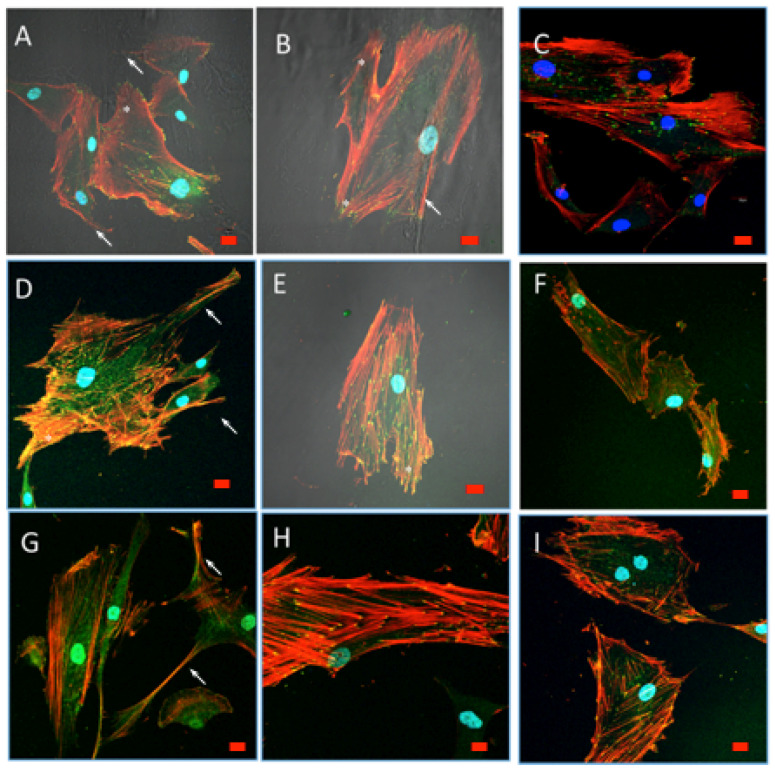
HOB^®^ osteoblasts grown on CS10G4 sample after 48 h (**A**,**B**), 72 h (**D**,**E**) and 1 week (**G**,**H**) in culture, and examined with confocal microscope (40× objective lens). Osteoblasts grown on glass were used as reference control, shown in (**C**), for 48 h in culture, (**F**) after 72 h in culture and (**I**) after 1 week. In red, rhodamine–phalloidin immunolabelled actin cytoskeletal fibers, showing polarization to material and actin cytoskeletal arrangement into stress fibers. Focal adhesions (yellow) were immunolabelled with antivinculin antibody. Nuclei (blue) were DAPI labelled. Arrow marks filopodial emissions, star marks lamellipodia. Scale bar represents 20 μm.

After 48 h in culture, cell spreading evolved to a near confluence stage, with well differentiated osteoblasts adhered to the surface and tethering contacts to the neighboring cells. Osteoblasts grown on bare glass, although well adhered, did not spread to confluence, showing discrete cell overlapping after 48 h in culture (Figure 9 and Figure 10). From 72 h onwards, cells grown on experimental substrata elongated, and cytoskeletal polarization increased with a significant number of mature focal adhesions linked to actin stress fibers. After 1 week Cells grown on CS10G4 appeared to be more elongated than control and CS7G.

#### 3.9.2. Cytoskeletal Organization and Focal Adhesions

**Figure 11:**
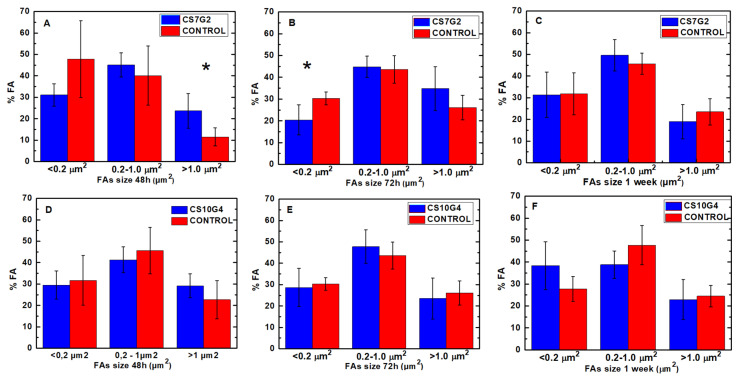
Percentage of FAs according to size. Comparative analysis between cells grown on CS7G2 and control groups at (**A**): 48 h hours in culture; (**B**): 72 h hours in culture; (**C**): 1 week in culture; and cells grown on CS10G4 and controls in (**D**): 48 h hours in culture; (**E**): 72 h hours in culture; (**F**): 1 week in culture (*: *p* < 0.005).

Actin cytoskeleton immunolabelling of growing cells revealed clear differences both in cell behavior and in cytoskeletal arrangement. Osteoblasts grown on CS7G2 sample were phenotypically elongated and clustered in a reticular pattern from 48 h in culture onwards. From 48 h onwards osteoblasts elongated with increased elongation and stress fibers development, and a more defined osteoblast orientation was found, together with a higher number of well-developed focal adhesions. While cells grown on glass developed scarce or no stress fibers, cells grown on CS7G2 and CS10G4 samples showed a significant increase in well-developed stress fibers and focal adhesions (*p* < 0.05), mainly evident after 1 week h in culture. Although both groups significantly differed from controls (*p* = 0.004 and *p* = 0.014, respectively) no significant differences were found between experimental groups along experimental times (Figure 9, Figure 10 and Figure 11).

The focal adhesion complexes consists of integrin and actins vertically separated by a core that includes cytoskeletal elements such as vinculin [60,61,62,63]. As shown, mature focal adhesions appear in experimental groups, initially on the tips of filopodia and in the leading edge lamellipodia after 72 h (Figure 9, Figure 10 and Figure 11). While in control cells a certain turnover can be appreciated, with small focal adhesions, in experimental groups the FA complexes maturated, together with a higher expression of stress fibers, as described by us and others [60,62,64,65,66], as an expression of reduced cell migration and of the initial steps cell differentiation. After 1 week the percentage of mature and intermediate size FAs increased, together with a well-developed stress fiber network including a significant number of mature focal adhesions, mainly in cells grown on CS10G4 samples.

Spatiotemporal regulation of tension sustained at FAs has been described as essential for regulation of cell migration and settlement pointing to extracellular matrix remodeling and new bone formation. Force-mediated FA signaling together with actin bundles organization in stress fibers regulates cell proliferation and differentiation. Both CS7G2 and CS10G4 have demonstrated to be bioactive and non-cytotoxic and, at the same time, induce focal adhesion formation and maturation of HOB^®^ cells in culture. In the presence of aerogels, osteoblasts change their morphology showing changes in cell morphology compatible with cell migration, such as filopodial and lamellipodial emissions towards material surface, at initial experimental times, reinforced with focal adhesion and stress fibers development.

According to our data, time dependent maturation of focal adhesion with significant presence of mature focal adhesion complexes and stress fiber development appears earlier in CS10G4 samples, while in cells grown in the presence of CS7G2 nascent and punctate focal adhesion points remaining for a longer period of time, located in periphery, thus indicating a sustained migration capability significantly higher than control cells and presumably induced by the biomaterial. This data are in agreement with the mechanical properties described above and, although the mechanical response in SBF is not as good as in dry conditions, the mechanoinductive properties of the biomaterial appear to be adequate.

Taken together, these results point to a positive effect on HOB^®^ cells of the proposed material in which both physicochemical and topographical properties appear to be involved [60,67,68,69,70,71,72].

## 4. Conclusions

CS-silica and CS-GPTMS-silica mesoporous hybrid aerogels with interconnected high porosity could be potentially used as substitute material for bone tissue regeneration. Monolith crack free specimens were obtained by sol-gel, followed by CO_2_ supercritical drying. The aerogels presented a fast uptake and swelling in PBS solution by fast imbibition, with swelling capacities ranging from 1.75 to 3.75 by decreasing the GPTMS content from 4 to 2 molar ratio with respect to CS monomer. Mechanical solicitation of class II hybrid crosslinked aerogels in swollen state in PBS, showed compressive strengths about 100–250 kPa, retaining their monolith geometry until fracture, thus exhibiting their potential efficacity to fill the empty space of bone defects. Further understanding of degradation kinetics to examine the time dependence of weight losses during soaking in PBS is required. Also the quantification of silicon release will be important to completely describe the hydrolytic degradation process of the hybrids, in order to perform this novel bioactive system for conducting hydrophilic therapeutic biomaterials to improve bone tissue engineering. Furthermore, the ability to induce and control the growth of a bioactive layer formed by HAp spherulites above 2 µm in diameter after 21 days of soaking in SBF promotes the adhesion and proliferation of osteoblasts, contributing effectively in the bone regeneration process. Although the exact relationship between focal adhesion maturation and traction forces role is not yet elucidated, our results clearly indicate that CS-GPTMS-silica hybrids are not only biocompatible and bioactive, without cytotoxically effects, but also induce cell adhesion, cytoskeletal rearrangement and elongation with stress fibers development due to the presence of mature focal adhesion complexes. The results described point to the materials above as good alternatives in bone tissue cells recruitment and maturation, inducing an excellent initial osteoblast response in vitro.

## Figures and Tables

**Table 1:** Sample identification, starting compositions and final content of CS based on Elemental Analysis (EA) measured data for each synthesized aerogel.

Sample Identification	Nominal CS Content	GPTMS/CS	TEOS/CS	Final CS Content from EA ^1^
	(wt%)	(Molar Ratio)	(Molar Ratio)	(wt%)
CS1G0	3.8	0	150	1.7
CS2G0	7.3		75	3.3
CS3G0	12.3		45	7.6
CS4G0	16.7		30	10.3
CS5G2	3.7	2	150	3.1
CS6G2	6.8		75	5.9
CS7G2	14.2		30	8.0
CS8G4	6.3	4	75	3.1
CS9G4	9.8		45	5.3
CS10G4	12.3		30	9.7

^1^ Final CS content was calculated from the N values measurements in EA, considering that chitosan is the sole source of nitrogen in the samples.

**Table 2:** Bulk density and textural data from N_2_ physisorption for CS-GPTMS-Silica hybrid aerogels.

Sample	Density (±0.01 g cm^−3^)	Physisorption			
		S_BET_^1^ (m^2^ g^−1^)	Pore Volume (cm^3^ g^−1^)	Pore Size (nm)	Porosity (%)
CS1G0	0.38	1184	2.6	8.1	81.9
CS2G0	0.26	1230	3.4	10.2	87.6
CS3G0	0.17	811	2.3	10.2	82.4
CS4G0	0.17	755	1.9	11.1	91.6
CS5G2	0.29	1042	3.1	11.2	86.1
CS6G2	0.22	903	3.2	14	89.3
CS7G2	0.23	771	2.2	11.6	89.1
CS8G4	0.31	955	2.1	8.7	85.4
CS9G4	0.32	697	1.6	8.9	82.6
CS10G4	0.37	695	1.7	9.8	82.3

^1^ Correlation coefficient for BET surface area measurement was higher than 0.9996.

**Table 3:** Mechanical properties obtained from the uniaxial compression testing of the dry samples and wet samples after saturation by the absorption of PBS for CS/GPTMS-Silica aerogels; mean values ± standard deviation (*n* = 3).

Sample	Young’s Modulus (MPa)		Compressive Strength (MPa)		Maximum Compressive Strain (%)	
	Dry	Wet	Dry	Wet	Dry	Wet
CS1G0	11.2 ± 1.4	1.13 ± 0.44	1.0 ± 0.3	0.17 ± 0.02	12.2 ± 2.3	13.20 ± 3.40
CS2G0	9.9 ± 2.9	0.30 ± 0.10	0.8 ± 0.2	0.07 ± 0.01	18.1 ± 3.8	13.47 ± 0.21.
CS3G0	1.9 ± 0.1	0.30 ± 0.07	16.1 ± 4.2	0.09 ± 0.05	66.4 ± 7.0	12.60 ± 1.71
CS4G0	2.8 ± 0.6	0.75 ± 0.25	1.46 ± 0.1	0.11 ± 0.04	50.4 ± 5.5	9.18 ± 1.55
CS5G2	21.1 ± 7.0	0.78 ± 0.07	5.3 ± 4.8	0.19 ± 0.05	36.5 ± 25.6	12.92 ± 1.30
CS6G2	7.7 ± 1.5	0.40 ± 0.01	22.6 ± 15.1	0.16 ± 0.01	77.6 ± 16.3	21.21 ± 2.09
CS7G2	6.7 ± 1.3	0.16 ± 0.02	77.7 ± 2.7	0.09 ± 0.03	77.7 ± 1.6	22.89 ± 9.47
CS8G4	32.4 ± 3.8	0.77 ± 0.30	5.6 ± 3.1	0.21 ± 0.01	35.2 ± 10.7	9.41 ± 3.01
CS9G4	8.7 ± 0.5	0.82 ± 0.17	5.5 ± 2.9	0.27 ± 0.04	38.0 ± 14.9	10.58 ± 0.86
CS10G4	50.3 ± 7.0	0.27 ± 0.03	95.7 ± 6.9	0.26 ± 0.03	76.2 ± 9.5	21.74 ± 1.37

## Supplementary Materials

The following are available online at https://www.mdpi.com/2073-4360/12/11/2723/s1, Figure S1. Image of CS/GPTMS-SiO2 hybrid aerogel monoliths with different GPTMS content obtained by CO_2_ supercritical drying. Left (CS4G0), centre (CS7G2) and right (CS10G4) samples containing 10.3, 8.0 and 9.7 wt% chitosan, respectively; Figure S2. (a) N2 physisorption isotherms of selected CS/GPTMS-SiO_2_ aerogels with different GPTMS/CS molar ratio and (b) their corresponding pore size distributions (PSD); Figure S3. Thermogravimetry TG and differential thermogravimetry DTG (inset) for CS1G0, CS4G0, CS7G2 and CS10G4 hybrid aerogels; Figure S4. Stress–strain curves for selected samples (a) uniaxial compression of dry aerogels, as taken from the autoclave; (b) uniaxial compression of corresponding wet aerogel samples saturated in PBS solution (w samples).
