# Supplementary material for: Chitosan-GPTMS-Silica Hybrid Mesoporous Aerogels for Bone Tissue Engineering
Source: Polymers (Basel). 2020 Nov 17;12(11):2723. doi: 10.3390/polym12112723 (PMC7698430; doi:10.3390/polym12112723)
Supplement: Supplementary file 1 [file polymers-12-02723-s001.pdf]

Chitosan-GPTMS-Silica hybrid mesoporous aerogels for bone tissue engineering

M.V. Reyes-Peces, A. Pérez-Moreno , D. M. de los Santos , M. Mesa-Díaz, G. Pinaglia-Tobaruela, JI. Vilches-Pérez, R. Fernández-Montesinos, M. Salido, N. de la Rosa-Fox and M. Piñero

■ SUPPLEMENTARY MATERIAL

SM.1 Image of hybrid aerogel monoliths prepared via supercritical drying in CO_2

The samples were obtained as well defined cylinders

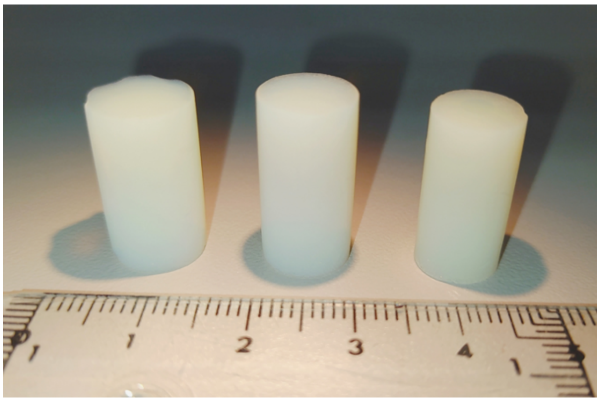

Figure S1. Image of CS/GPTMS-SiO2 hybrid aerogel monoliths with different GPTMS content obtained by CO2 supercritical drying. Left (CS4G0), centre (CS7G2) and right (CS10G4) samples containing 10.3, 8.0 and 9.7 wt. % chitosan, respectively

SM.2 Nitrogen physisorption experiments. Analysis of the mesopore network structure

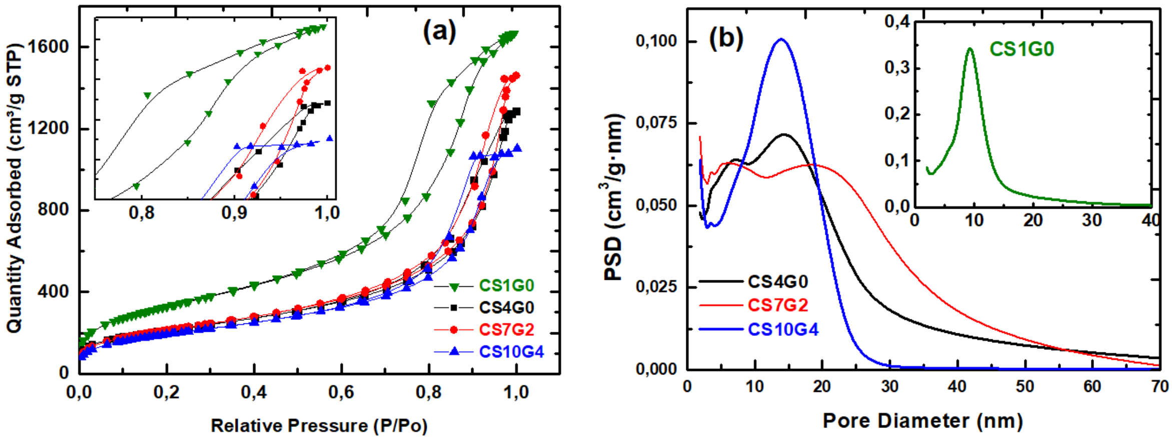

Figure S2. (a) N_2 physisorption isotherms of selected CS/GPTMS-SiO2 aerogels with different GPTMS/CS molar ratio and (b) their corresponding pore size distributions (PSD)

SM.3 Thermal Analysis

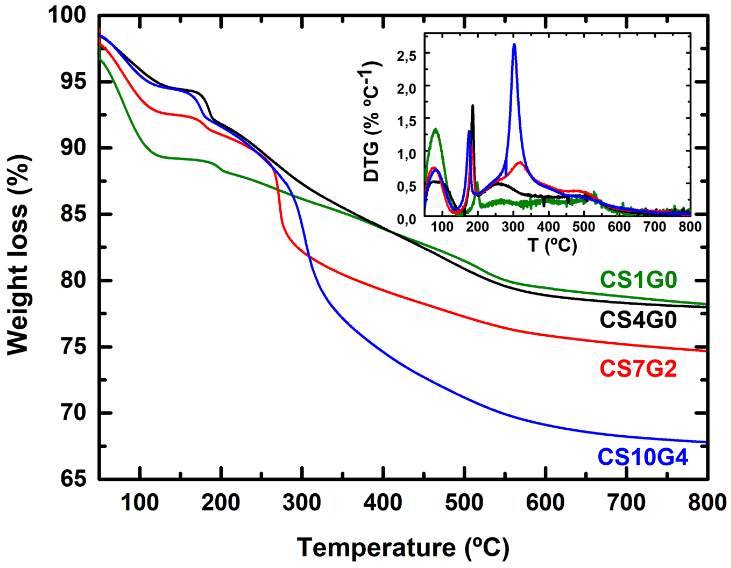

Figure S3. Thermogravimetry TG and differential thermogravimetry DTG (inset) for CS1G0, CS4G0, CS7G2 and CS10G4 hybrid aerogels.

SM.4 Mechanical properties

Fig. S4 shows the stress-strain curves from uniaxial compression of samples CS1G0, CS4G0, CS7G2 and CS10G4 in dry state (Fig. S4a) and saturated by liquid PBS (Fig. S4b). It can be seen how the GPTMS enhances the mechanical response, due to the existence of a covalent crosslinking hybrid network. The experiment performed in liquid PBS yields similar behaviour for both samples type, with and without crosslinker, decreasing their compression strength for about 96 fold.

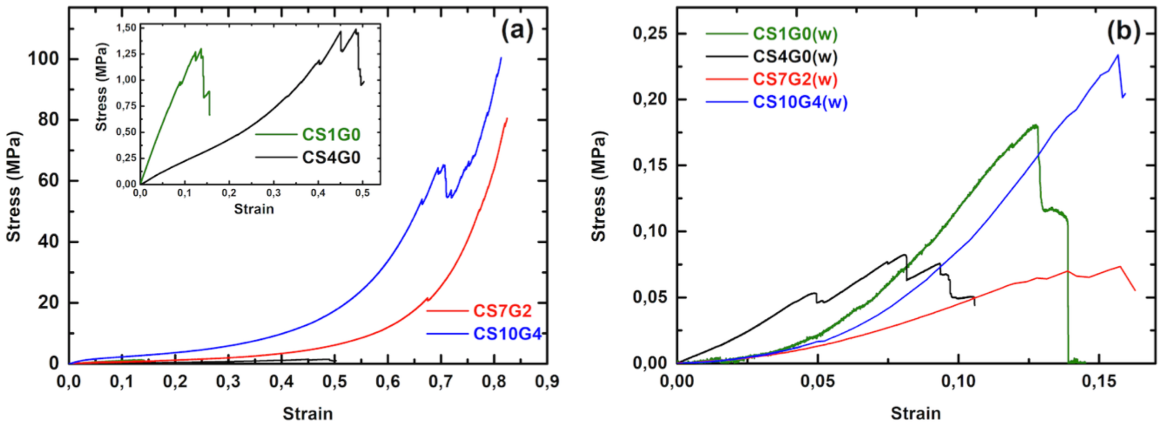

Figure S4. Stress–strain curves for selected samples (a) uniaxial compression of dry aerogels, as taken from the autoclave; (b) uniaxial compression of corresponding wet aerogel samples saturated in PBS solution (w samples).
